# microRNA-7 as a tumor suppressor and novel therapeutic for adrenocortical carcinoma

**DOI:** 10.18632/oncotarget.5383

**Published:** 2015-10-01

**Authors:** Anthony R. Glover, Jing Ting Zhao, Anthony J. Gill, Jocelyn Weiss, Nancy Mugridge, Edward Kim, Alex L. Feeney, Julian C. Ip, Glen Reid, Stephen Clarke, Patsy S.H. Soon, Bruce G. Robinson, Himanshu Brahmbhatt, Jennifer A. MacDiarmid, Stan B. Sidhu

**Affiliations:** ^1^ Cancer Genetics Laboratory, Kolling Institute, Northern Sydney Local Health District, St Leonards, NSW, Australia; ^2^ Sydney Medical School Northern, Royal North Shore Hospital, University of Sydney, St Leonards, Sydney, NSW, Australia; ^3^ Department of Anatomical Pathology, Royal North Shore Hospital and University of Sydney, St Leonards, Sydney, NSW, Australia; ^4^ EnGeneIC Ltd, Lane Cove West, Sydney, NSW, Australia; ^5^ Asbestos Diseases Research Institute, University of Sydney, Concord, Sydney, NSW, Australia; ^6^ Department of Oncology, Royal North Shore Hospital and University of Sydney, St Leonards, Sydney, NSW, Australia; ^7^ Ingham Institute for Applied Medical Research, University of New South Wales, Liverpool, NSW, Australia; ^8^ Department of Endocrinology, Royal North Shore Hospital and University of Sydney, St Leonards, Sydney, NSW, Australia; ^9^ University of Sydney Endocrine Surgery Unit, Royal North Shore Hospital, Sydney, St Leonards, Sydney, NSW, Australia

**Keywords:** noncoding RNA, miR-7, nanoparticle therapy, adrenal cancer

## Abstract

Adrenocortical carcinoma (ACC) has a poor prognosis with significant unmet clinical need due to late diagnosis, high rates of recurrence/metastasis and poor response to conventional treatment. Replacing tumor suppressor microRNAs (miRNAs) offer a novel therapy, however systemic delivery remains challenging. A number of miRNAs have been described to be under-expressed in ACC however it is not known if they form a part of ACC pathogenesis. Here we report that microRNA-7–5p (miR-7) reduces cell proliferation *in vitro* and induces G_1_ cell cycle arrest. Systemic miR-7 administration in a targeted, clinically safe delivery vesicle (^EGFR^EDV^TM^ nanocells) reduces ACC xenograft growth originating from both ACC cell lines and primary ACC cells. Mechanistically, miR-7 targets Raf-1 proto-oncogene serine/threonine kinase (RAF1) and mechanistic target of rapamycin (MTOR). Additionally, miR-7 therapy *in vivo* leads to inhibition of cyclin dependent kinase 1 (CDK1). In patient ACC samples, *CDK1* is overexpressed and miR-7 expression inversely related. In summary, miR-7 inhibits multiple oncogenic pathways and reduces ACC growth when systemically delivered using EDV^TM^ nanoparticles. This data is the first study in ACC investigating the possibility of miRNAs replacement as a novel therapy.

## INTRODUCTION

The revelation that non-coding RNAs have diverse regulatory functions has caused a revolution in the understanding of biology [1]. In little of over a decade since the discovery that loss of microRNA (miRNA) expression is associated with chronic lymphocytic leukemia [2], it is now known that miRNA dysregulation extends to all cancers and that miRNAs are regulators of gene expression [3]. As miRNAs can function as tumor suppressors and target multiple cell pathways they have potential as novel therapeutic agents [4]. One cancer in which novel therapeutics is needed is adrenocortical carcinoma (ACC). ACC is a rare but aggressive cancer with limited treatment options and an overall 5-year survival of less than 35% for metastatic disease [5]. This need for new treatments has been highlighted by the poor outcomes from the FIRM-ACT trial [6], where conventional treatment for metastatic ACC—Mitotane in combination with Etoposide, Doxorubicin and Cisplatin was found to have a response rate of only 23%. If effective and safe, miRNA therapy could offer a new treatment modality. For miRNA replacement therapy to be rational, the miRNA should be shown to be deficient in the cancer and have a functional basis for an anti-cancer effect such as targeting known cancer related pathways. One miRNA deficient in ACC is microRNA-7–5p (miR-7) [7]. In an earlier study of miRNA expression in adrenocortical tumors from our group, miR-7 was the most significantly under-expressed miRNA with the largest decrease in fold change (18 ×) compared to expression in normal adrenal cortex (NAC) [7].

miRNAs function as 21–24 nucleotide guides that regulate the expression of mRNAs containing complementary sequences by direct mRNA destabilization, however miRNA complexes also use other mechanisms to block protein expression [8] and induce both direct and indirect transcriptional changes [9]. The challenge for miRNA therapy for cancer is to establish safe and effective delivery systems. These systems would ideally have specificity for cancer cells and reduce uptake by non-cancer cells to avoid off-target effects. A number of different delivery vehicles have been investigated including polypeptides, adenovirus and lipids however most of these systems have yet to be shown to be safe for human use [10]. Despite these challenges two delivery systems have reached the clinic and are currently under assessment in cancer clinical trials using liposome formulated miR-34a for liver cancer [11] and miR-16 packaged in genetically modified bacterial minicell particles called EnGeneIc Delivery Vehicles (EDVs) for mesothelioma [12, 13].

In this study we show that miR-7 acts as a tumor suppressor in ACC and systemic delivery of miR-7 using EDV nanoparticles is effective in reducing tumor growth in both cell line and primary culture ACC xenograft models. We demonstrate that miR-7 therapy leads to repression of multiple genes involved in the pathogenesis of ACC, including Raf-1 proto-oncogene serine/threonine kinase (*RAF1*), mechanistic target of rapamycin (*MTOR*) and cyclin dependent kinase 1 (*CDK1*). This is the first study in ACC, presenting pre-clinical proof-of-concept evidence for the utility of miRNA replacement as a therapeutic strategy.

## RESULTS

### miR-7 is under-expressed in ACC clinical samples

**Figure 1:**
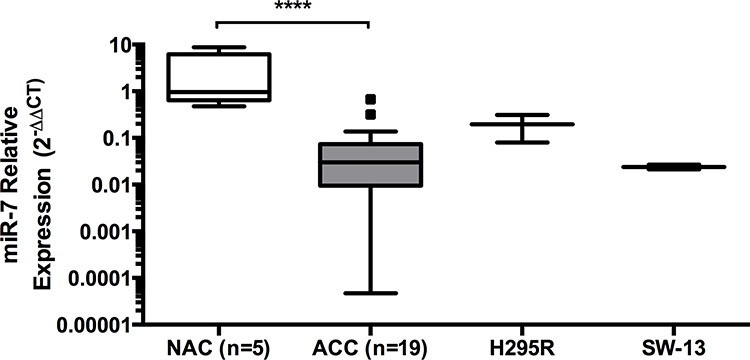
miR-7 is under-expressed in ACC clinical samples miR-7 expression was under-expressed in clinical samples of ACC compared to normal adrenal cortex (NAC), reduced miR-7 expression was found in the ACC cell lines H295R and SW-13 (3 replicates shown), *RNU48* reference gene, median expression shown, data presented as Tukey Box Plot, median expression is represented by the solid line within the box shown, and true outliers (>1.5 × interquartile range) are represented by the dots outside the boxes, ****indicates *P* < 0.0001.

We have previously shown by miRNA microarray analysis, that low expression of miR-195 and miR-483–5p are associated with poor disease-specific survival in ACC [7]. In that study, miR-7, while not being associated with outcome, was identified as the most significantly down-regulated miRNA in a cohort of clinical ACC samples; miR-7 was reduced by 18-fold in ACC compared to NAC (*P* = 6.15E-10) [7]. To confirm this finding, RT-qPCR was performed on a different group of 19 ACC and 5 NAC clinical samples. This analysis showed that miR-7 was significantly reduced in ACC compared to NAC with a fold change of 0.04 or a 25-fold reduced expression of miR-7 in ACC (Figure 1). ACC has two established cells lines available (H295R and SW-13) both of which also had reduced miR-7 expression compared to the NAC samples (Figure 1). H295R cells are hormone producing (functional) and the best characterized in study of the ACC, while SW-13 are non-hormone producing and while derived from an adrenal surgical sample it is not clear whether they arose from a primary ACC or metastasis [14]. For this study, SW-13 cells were used as secondary cell line to investigate miR-7 action in a non-functional ACC model.

### miR-7 inhibits cell proliferation and induces cell cycle arrest

**Figure 2:**
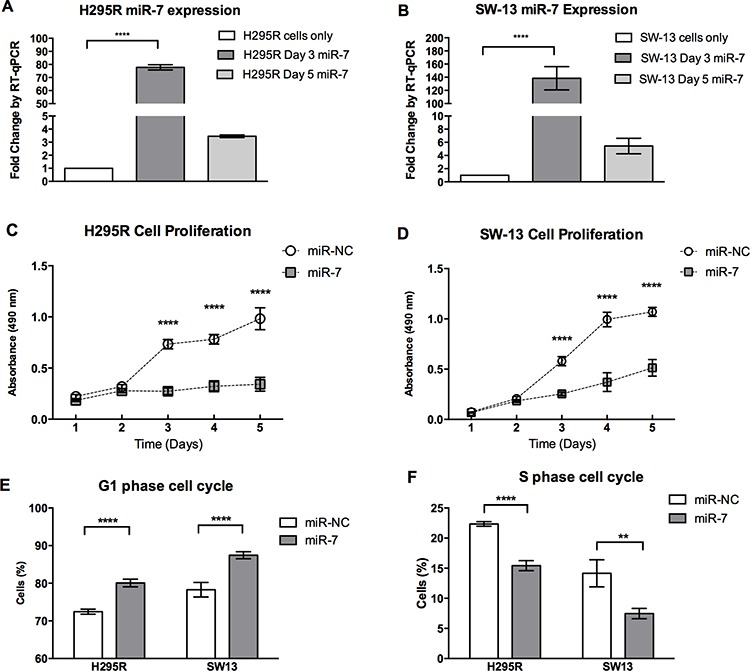
miR-7 inhibits cell proliferation and induces cell cycle arrest **A, B.** Expression levels of miR-7 in cell lines H295R and SW13 three and five days after transfection with miR-7 mimic. *RNU48* reference gene, error bars show SEM, **** indicates *P* < 0.0001. **C, D.** H295R and SW-13 cell proliferation was reduced following miR-7 replacement compared to miR-NC. Cell proliferation was assessed using MTS assays by three experiments, error bars show SEM, ****indicates *P* < 0.0001. **E, F.** Following miR-7 replacement, G_1_ phase of cell cycle was increased by a mean 7.6% in H295R cells and increased by a mean 9.2% in SW-13 cells in miR-7 treated cells compared to miR-NC treated cells. S phase was reduced by a mean 6.9% in H295R cells and reduced by a mean 6.7% in SW-13 cells in miR-7 treated cells compared to miR-NC treated cells. Cell cycle was assessed using flow cytometry with PI staining by three experiments. ** indicates *P* < 0.01, **** indicates *P* < 0.0001, error bars show SEM.

To explore the role of endogenous miR-7 in the pathogenesis of ACC, miR-7 was over-expressed in the H295R and SW-13 cells and the cell phenotypes induced were studied using scrambled miRNA sequences (miR-NC) as a negative control. Following transfection, increased miR-7 expression was confirmed by RT-qPCR (Figure 2A, 2B). Over-expression of miR-7 resulted in significant inhibition of cell proliferation in both H295R and SW-13 cells (Figure 2C, 2D). The cause of the reduction in cell proliferation following miR-7 overexpression was explored using analysis of cell division and cell death. Cell division analysis using flow cytometry showed cells transfected with miR-7 had a significantly reduced percentage in S phase and an increase in G_1_ phase when compared to miR-NC transfected cells (Figure 2E, 2F). Cell death analysis using apoptosis assays revealed no significant changes in the cell population transfected with miR-7 vs. miR-NC (data not shown). From these results, we propose that miR-7 in ACC acts *in vitro* to reduce cell proliferation by inducing G_1_ cell cycle arrest.

### RAF1 and MTOR are reduced following miR-7 replacement in ACC cell lines

To investigate the mechanisms through which miR-7 may act as a tumor suppressor, we examined predicted targets of miR-7 using four prediction algorithms; DIANA-microT-CDS v5.0 (B.S.R.C. Alexander Fleming, Athens, Greece), DIANA-miRPath v2.0 (B.S.R.C. Alexander Fleming), TargetScan (Whitehead Institute for Biomedical Research, MA, USA) and miRanda (Welcome Trust Sanger Institute, UK). The analysis of DIANA-miRPath v2.0 predicted that mammalian target of rapamycin (mTOR) signalling pathway as the top pathway targeted by miR-7 with 10 genes, including mechanistic target of rapamycin (*MTOR*) and eukaryotic translation initiation factor 4E (*EIF4E*). A list of predicted targets from each database, which encode pivotal components of key cancer-related signalling pathways were compared and intersected to narrow the list of potential genes to higher confidence targets. Selected predicted targets for experimental validation are listed in Supplementary Table 1. Targets selected included *RAF1* and Epidermal Growth Factor Receptor (*EGFR*) as key genes in the mitogen-activated protein kinase pathway (MAPK) signalling pathway and *MTOR* and *EIF4E* involved in the mTOR pathways.

**Figure 3:**
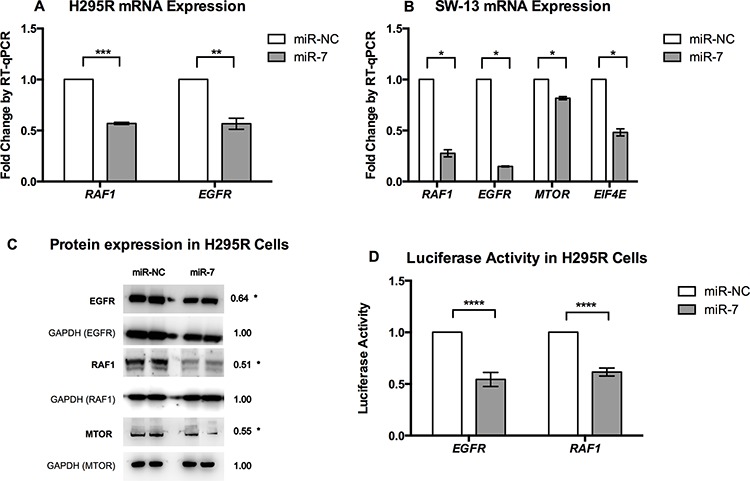
*RAF1* and *MTOR* are reduced following miR-7 replacement in ACC cell lines **A.** Following miR-7 replacement in H295R cells, mRNA levels of *RAF1* and *EGFR* were reduced. *GAPDH* reference gene, error bars show SEM, ** indicates *P* < 0.01, *** indicates *P* < 0.001. **B.** Following miR-7 replacement in SW-13 cells, reduced mRNA levels of *RAF1*, *EGFR*, *EIF4E* and *MTOR* were detected. *GAPDH* reference gene, error bars show SEM, * indicates *P* < 0.05. **C.** Following miR-7 replacement in H295R cells, reduced mean protein expression of EGFR, RAF1 and MTOR was detected compared to miR-NC treated cells. Representative images of one experiment shown, number refers to mean densitometry measurement taken from three experiments. * indicates *P* < 0.05. **D.** Co-transfection of the luciferase-reporter vector containing 3′ UTR of *EGFR* and *RAF1*, respectively along with miR-7 mimics suppressed luciferase activity. Assessed by three experiments, mean luciferase activity shown and adjusted to miR-NC activity = 1 for both vectors, error bars show SEM, **** indicates *P* < 0.0001.

To determine whether miR-7 repressed any of these putative targets, cells were transfected and mRNA levels assessed by RT-qPCR. Over-expression of miR-7 significantly reduced expression of *RAF1* and *EGFR* in both H295R and SW-13 ACC cell lines (Figure 3A, 3B), while *MTOR* and *EIF4E* was significantly reduced in SW13 cells only (Figure 3B). Reduced expression of RAF1, EGFR and MTOR protein by miR-7 was detected by Western blotting in H295R cells (Figure 3C). Both *RAF1* and *EGFR* contain two predicted seed binding sites in their 3′ UTRs that are highly conserved in mammals (Supplementary Figure 1). To verify that these transcripts are directly regulated by miR-7, the 3′ UTR sequences of *EGFR* and *RAF1*, encompassing the predicted seed binding sites that would disrupt miRNA interaction, were inserted into the multiple cloning site of the pMIR-REPORT miRNA Expression Reporter Vector (Life Technologies). Co-transfection of the reporter vector with miR-7 mimic or miR-NC was performed in H295R cells. Co-transfection with miR-7 suppressed luciferase activity of both the *RAF1* and *EGFR* reporters (Figure 3D) when compared with that co-transfected with miR-NC, confirming the seed binding sequences of miR-7 on the 3′ UTR region of *RAF1* and *EGFR* genes. Taken together, these results indicate miR-7 acts as a tumor suppressor in ACC by affecting multiple molecular targets, involved in the mTOR and MAPK signalling pathways.

### Therapeutic miR-7 replacement using EDV nanoparticles reduces ACC xenograft growth

**Figure 4:**
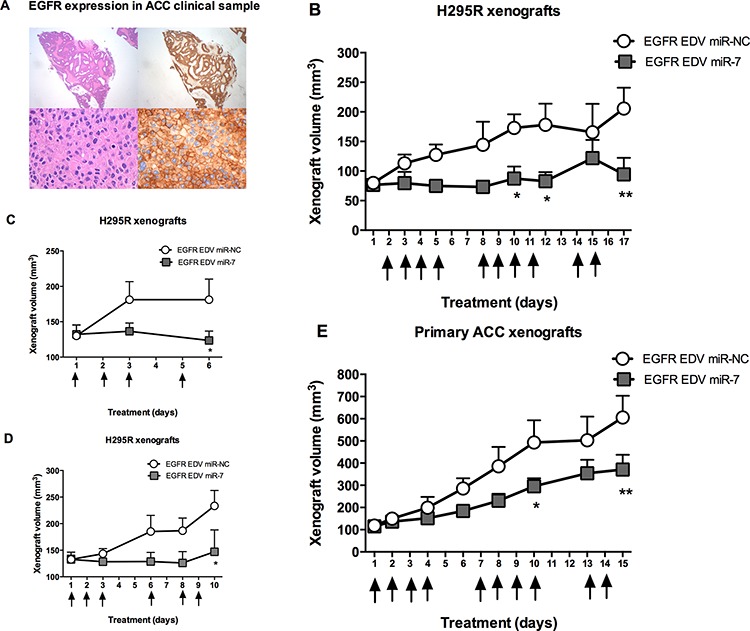
Targeted miR-7 replacement using EDV nanoparticles reduces ACC cell line and patient derived xenograft growth **A.** EGFR is expressed in ACC, image shows H&E (left) and EGFR (right) stained sections of ACC in patient sample showing diffuse strong EGFR positivity across the tumor, 20× magnification in top images & 600× magnification in bottom images. **B.** H295R xenografts in (*nu/nu*) mice were treated with systemic ^EGFR^EDV^TM^ nanocells containing either miR-7 or miR-NC (*n* = 6 for each group). Arrows indicate days of treatment, mean volumes shown for each group, error bars show SEM, * indicates *P* < 0.05, ** indicates *P* < 0.01. **C, D.** Repeat experiment of H295R xenografts in (*nu/nu*) mice treated with four doses and six doses of systemic ^EGFR^EDV^TM^ nanocells respectively containing either miR-7 or miR-NC (*n* = 6 for each group), mean volumes shown for each group, error bars represent SEM, * indicates *P* < 0.05. **E.** Primary ACC xenografts were established in (*nu/nu*) mice and treated with ^EGFR^EDV^TM^ nanocells containing either miR-7 or miR-NC (*n* = 5 for each group). Arrows indicate days of treatment, mean volumes shown for each group, error bars show SEM, * indicates *P* < 0.05, ** indicates *P* < 0.01.

Having demonstrated that miR-7 arrests proliferation of ACC cells *in vitro*, we next initiated a series of *in vivo* experiments to assess whether targeted delivery by intravenous injection of miR-7 using EDV nanoparticles could be used as a therapeutic for ACC. H295R and patient-derived xenograft models were established and miR-7 mimic was delivered using the targeted nanoparticle delivery system—^EGFR^EDV^TM^ nanocells [12]. EGFR targeted nanoparticles were used as EGFR is expressed in ACC (Figure 4A).

For the initial H295R xenograft experiment, miR-7 and miR-NC was intravenously administered by tail vein injection at a dose of 2 × 10^9^ EDVs containing 0.32 nmoles of either miR-7 or miR-NC in ten doses over a two-week period. After 17 days, ACC tumor volume had increased by over two fold in the miR-NC treated group and remained unchanged in the miR-7 treated group (Figure 4B). To confirm these findings and to investigate the molecular target knock down of miR-7 regulation of molecular targets, H295R xenografts were established on two further separate occasions and tumors were collected following four and six doses of treatment. miR-7 therapy demonstrated tumor reduction as early as two doses of treatment and similar tumor inhibition effect was seen with each experiment (Figure 4C, 4D).

To further test the use of miR-7 replacement as a therapeutic, we tested this regimen in a patient derived xenograft. ACC primary cells were isolated from an ACC surgical sample and inoculated subcutaneously. Systemic delivery of ten doses of miR-7 in this xenograft model showed significant tumor reduction in the miR-7 group vs. miR-NC group at the end of the treatment period (Figure 4E). Taken together, we have demonstrated that systemic targeted miR-7 replacement using a nanoparticle delivery system inhibits ACC growth in both cell line and patient-derived xenografts.

### Systemic miR-7 therapy *in vivo* leads to inhibition of *RAF1*, *MTOR* and *CDK1*

**Figure 5:**
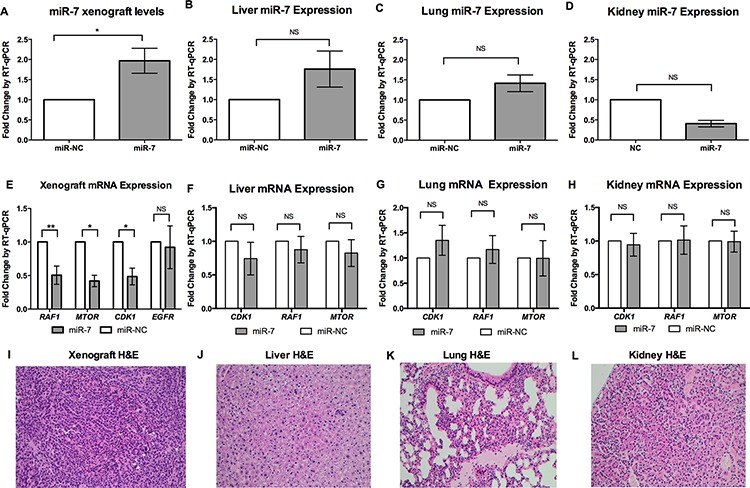
Systemic miR-7 therapy *in vivo* leads to inhibition of *RAF1*, *MTOR* and *CDK1* without evidence of off-target effects **A, B, C, D.** Following miR-7 replacement, increased levels of miR-7 were found in H295R xenografts (*n* = 6 for each group) while no significant difference was seen in organs, *RNU48* reference gene for xenografts and mouse U6 snRNA reference gene for mouse organs, error bars show SEM, * indicates, *P* < 0.05, NS indicates *P*-value not significant. **E, F, G, H.** Following six doses of miR-7 replacement in H295R xenografts, reduced mRNA levels of *RAF1, MTOR* and *CDK-1* were detected, no significant difference in *EGFR* expression was detected, no significant difference of *CDK1, RAF1* or *MTOR* were detected in mouse liver, lung or kidneys compared to the miR-NC treated xenografts, *GAPDH* reference gene for xenografts, mouse *B2m* reference gene for mouse organs, error bars show SEM, ** indicates *P* < 0.01, * indicates *P* < 0.05, NS indicates *P*-value not significant. **I, J, K, L**: H&E. staining shown for miR-7 treated mice showed no difference in the xenograft, liver, lung or kidney compared to the miR-NC, H&E staining shown for miR-7 treated mice, 200× magnification.

**Figure 6:**
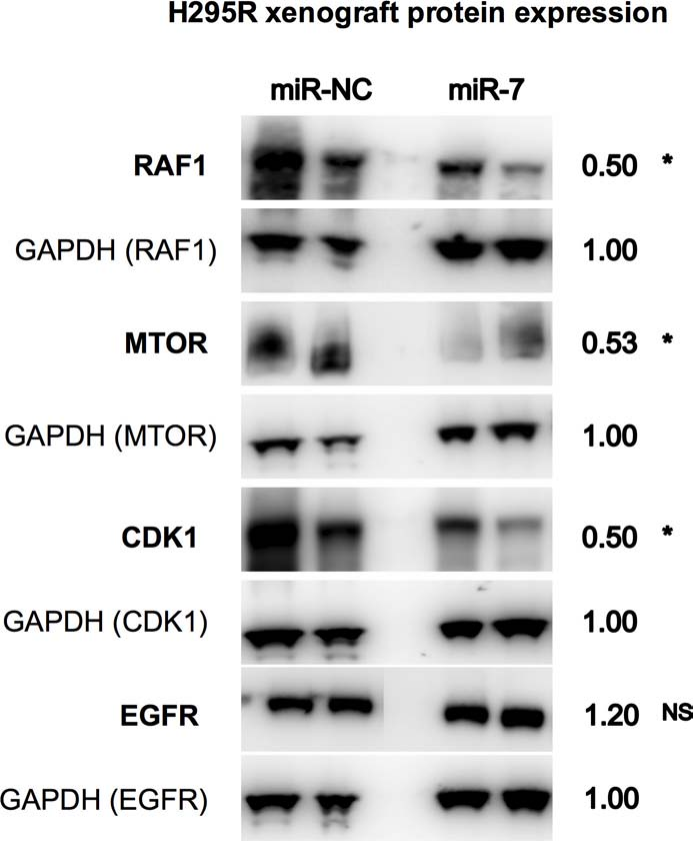
Reduced protein expression of RAF1, MTOR and CDK1 in mouse xenografts following miR-7 therapy Following six doses of miR-7 replacement in H295R xenografts, reduced mean protein expression of RAF1, MTOR and CDK1 was detected compared to miR-NC treated xenografts. Representative images of two xenografts per group shown, number refers to mean densitometry measurement taken from five xenograft samples. * indicates *P* < 0.05, NS indicates *P* value not significant.

To assess how miR-7 replacement reduces ACC xenograft growth, we first confirmed that the EDVs delivered miR-7 to the tumor cells. RT-qPCR was performed on excised xenografts and showed significantly increased miR-7 expression in miR-7 treated xenografts compared to those treated with miR-NC following six doses of EDVs (Figure 5A). Further to this, in the miR-7 treated xenografts both *RAF1* and *MTOR* were significantly down-regulated by over 2-fold (Figure 5E), with reduced protein expression of RAF1 and MTOR detected by Western blotting (Figure 6). However, no reduction of *EGFR* expression in the xenograft was detected using RT-qPCR, or Western blotting (Figure 5E, 6). In addition EGFR expression measured by immunohistochemistry also showed no change between miR-7 and miR-NC treated xenografts (data not shown). Histopathology showed similar tumor morphology between the miR-7 (Figure 5J) and miR-NC treated groups (data not shown).

In addition this analysis also detected significant down-regulation of cyclin-dependent kinase 1 (*CDK1*) in the miR-7 treated xenografts by RT-qPCR and Western blotting (Figure 5B, 6). *CDK1*, not being a predicted target of miR-7 was analysed due to the results of an earlier microarray study. In this study which was initially used to study long noncoding RNA and mRNA expression in ACC, differential expressed mRNAs between ACC vs. NAC clinical samples were analysed to identify genes that may be active in ACC [15]. In addition using Gene Set Enrichment Analysis (GSEA) [16] with KEGG pathway focus (Kanehisha Laboratories, Kyoto, Japan) the cell cycle pathway (KEGG Pathway ID: hsa04110) was found to be the highest enriched up-regulated pathway with 18 genes being significantly over-expressed in ACC vs. NAC (Enrichment score 7.65, *P*-value = 5.9 × 10–6). Five of these genes, including *CDK1*, Pituitary Tumour-Transforming 1 (*PTTG1*), cyclin B2 (*CCNB2*), cyclin E1 (*CCNE1*) and S-phase kinase-associated protein 2 (*SKP2*), were chosen to test whether these active genes in ACC may be inhibited following miR-7 therapy.

### *In vivo* off-target effect assessment

As with any new treatment modality, the possibility of side effects must be considered. While EDV nanocells have been assessed and found to be safe for human use in phase 1 clinical trials when delivering doxorubicin for recurrent glioma [17], the possibility of side effects from miR-7 itself has not been assessed. We examined the mouse liver, lungs and kidneys by histopathology, miR-7 expression and the expression of molecular targets repressed in the xenografts.

During our study period there was no significant difference in mouse weight between groups and no abnormal behavior or signs of toxicity were seen. No significant change of miR-7 was detected in liver, lung and kidney (Figure 5B, 5C, 5D) in the miR-7 treated mice in contrast to the significantly increased expression of miR-7 in the xenografts (Figure 5A). For the molecular endpoints reduced in the xenografts (*RAF1, MTOR* and *CDK1*), no significant difference could be seen in the liver, lung or kidney of mice treated with miR-7 compared to miR-NC (Figure 5F, 5G, 5H). H&E staining on each treated lung, liver and kidney, showed no difference between miR-7 and miR-NC treated groups with normal appearing organs for each treatment group (Figure 5J, 5K, 5L).

### In ACC patient samples, miR-7 expression is inversely associated with *CDK1* expression

**Figure 7:**
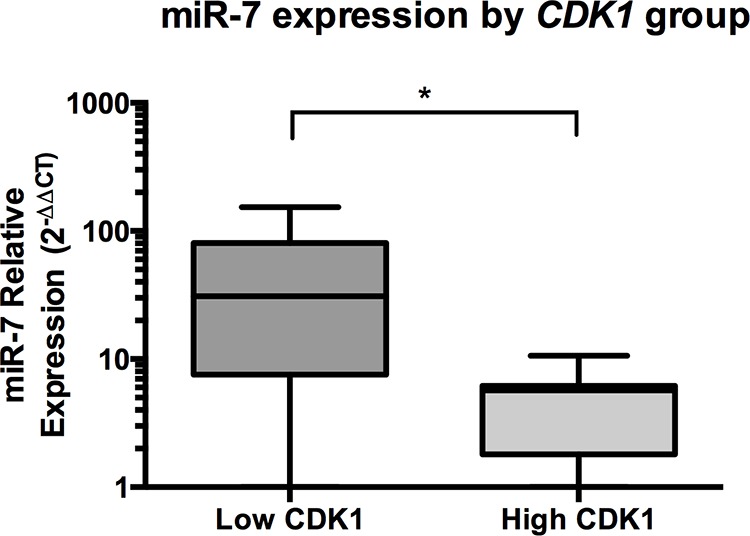
In ACC patient samples, miR-7 expression is inversely associated with CDK1 expression Median miR-7 expression was higher with low *CDK1* expression compared to high *CDK1* expression in ACC clinical samples (*n* = 15), high and low CDK1 expression was defined by a sample splitting method by median CDK1 expression of the clinical samples, data presented as Tukey Box Plot, median expression is represented by the solid line within the box shown, there were no true outliers (>1.5 × interquartile range), * indicates *P* < 0.05.

As part of our groups earlier study [15], using whole genome microarray analysis we found that *CDK1* was the most significantly over-expressed gene with the largest fold change 10-fold (*P* = 0.02) in clinical samples of ACC (*n* = 10) compared to normal adrenal cortex. To further investigate a potential functional relationship between miR-7 and *CDK1* we analysed the expression of these RNAs in an extended group of ACC clinical samples (*n* = 15) using RT-qPCR. Comparing *CDK1* and miR-7 expression analysis by scatter plot did not show a significant linear relationship. However, using a sample splitting method dividing *CDK1* expression into high and low groups (by median *CDK1* expression), we found the high *CDK1* expression group was associated with a significant lower expression of miR-7 (*P* = 0.04, Figure 7).

## DISCUSSION

This study demonstrates the potential of miRNA replacement as a therapeutic approach in ACC. We have shown that miR-7 acts as a tumor suppressor in ACC and that miR-7 replacement therapy reduces ACC xenograft growth. Restoration of miR-7 *in vitro* reduces cell proliferation and induces G_1_ cell cycle arrest. miR-7 replacement *in vivo* inhibits ACC xenograft growth in models derived from both H295R and primary ACC cells. miR-7 achieves this by directly targeting the MAPK (RAF1) and mTOR signalling pathways (MTOR), leading to inhibition of CDK1.

This study highlights the advantage of miRNA replacement over other RNA therapies such as short interfering RNAs in that miRNAs can target multiple cellular pathways [10] as demonstrated by the observation that miR-7 therapy down-regulates both the mTOR and MAPK pathways. The advent of targeted molecular therapies initially gave much hope for improvement in the outcomes of ACC. However it quickly became apparent that single agent therapies lead to treatment failures due to the activation of alternate tumor signalling pathways [18]. This failure was emphasised by the lack of efficacy with mTOR targeted therapies in ACC [18], where activation of other proteins such as Akt occurred [19]. In contrast, miRNAs offer a way to target multiple pathways, as demonstrated in our study, and thus have the potential to improve response rates. The MAPK and mTOR pathways are documented targets in ACC [18, 20] and with the added benefit of CDK1 knockdown we have seen with miR-7 *in vivo* therapy, this treatment offers the potential to improve ACC outcomes.

Our finding that miR-7 reduces xenograft tumor growth in both cell lines and patient derived cells is also of great interest. It is well recognised that xenograft models derived from cell lines, due to the selective pressures of cell culture can have poor predictive power in translation of findings to the clinic [21]. The use of patient-derived xenografts have been suggested as way to improve this predictive power [22] and it is encouraging that similar growth inhibition was seen with both our cell line and patient derived models.

miR-7 has been implicated as a tumor suppressor in multiple cancers including those associated with poor outcomes such as glioblastoma [23], non-small cell lung cancer [24] and gastric cancer [25] as well as other endocrine related cancers such as breast, prostate and ovarian cancer [26]. In breast cancer miR-7 has been identified as a tumor suppressor due to the interaction between the *miR-7–1* gene promoter and the transcription factor HoxD10 [27]. In addition, it has been suggested that miR-7 plays a role in the development of resistance to endocrine therapy by regulation of EGFR expression [28]. miR-7 also plays a role in physiological endocrine pathways, having been shown to play a regulatory role in insulin secretion in pancreatic islet cells [29]. miR-7 expression is also reduced in adrenocortical adenomas (ACA). Soon et al. found that miR-7 expression was low in ACA clinical samples compared to both NAC and ACC [7]. He et al. confirmed the finding of reduced miR-7 expression in ACAs compared to NAC and also compared to adrenocortical hyperplasia [30]. This findings support that reduced miR-7 expression may also play a role in ACA development and be an early change of adrenocortical tumor development which would be of interest for further study.

The finding that miR-7 can induce G_1_ cell cycle arrest has also been seen in hepatocellular carcinoma [31] and colorectal cancer [32]. Our *in vitro* studies showed a modest effect on G1 arrest being 7.6% for H295R cells and 9.3% for SW-13 cells but a significant reduction in cell proliferation in both cell lines (Figure 2). Similar findings have been demonstrated in hepatocellular carcinoma, where miR-7 induced a significant reduction in cell proliferation with similar rates of G1 arrest [33] and with let-7 in lung cancer [34]. The cause of this miR-7 induced G_1_ cell cycle arrest has being investigated in Chinese Hamster Ovarian cells, which identified multiple mRNAs involved in cell cycle regulation, including *CDK1* to be differentially expressed [35]. Our *in vitro* and *in vivo* findings support these results. Given our clinical findings of *CDK1* being over-expressed in ACC samples, plus the inverse correlation between *CDK1* and miR-7 expression, the *in vivo* inhibition of *CDK1* by targeted miR-7 delivery provides a strong rationale for miR-7 replacement as a potential novel therapeutic strategy.

As a target of miR-7 is *EGFR* mRNA and the ^EGFR^EDV nanocells are targeting EGFR it may seem counterintuitive to use this delivery system. However, while we detected EGFR knockdown *in vitro*, we were not able to see any significant change in EGFR expression *in vivo* by RT-qPCR, Western blotting or immunohistochemistry. The cause for these different results are not clear, however it may be possible that EGFR repression is achieved at higher levels of miR-7 delivery *in vitro* with Lipofectamine transfections compared to the nanoparticle delivery *in vivo*. A further explanation could be due to miR-7 reducing but not abolishing the expression of its targets, that a considerable reduction in EGFR expression was not achieved *in vivo* in the context of an established adrenal cancer xenograft with strong expression of EGFR.

Our finding of no significant off target effect from miRNA therapy is consistent with other published studies [11, 36, 37]. In the pre-clinical studies of miR-34a liposome delivery to liver tumors, analysis of the surrounding normal liver, lung, spleen and kidney showed increased miR-34a expression suggesting delivery to these organs, however no adverse effects were detected in the treated mice [11]. This work was used to advance a clinical trial which is currently recruiting patients in the USA and Korea and is due for completion in December 2015. Predicting the possible off-target effects of an introduced miRNA is difficult. This has been demonstrated in the difficulty to predict off target effects using sequence prediction with siRNAs. Hanning et al. have shown that siRNAs with a greater number of predicted hits do not necessarily produce more transcriptional off target effects and only 20.5% of transcriptional off target effects can be predicted [38]. Although more thorough assessment of off-target effects needs to be studied in future clinical pilot studies, our findings of no significant change of miR-7 levels and target expression in the treated liver, lungs and kidneys provide initial evidence of minimal off-target effect for systemic targeted miR-7 replacement therapy to provide support for potential human clinical trials.

Of great clinical potential, miR-7 has also been found to increase sensitivity to conventional therapeutics. In head and neck cancer cell lines, miR-7 overexpression has been shown to act synergistically with the tyrosine kinase inhibitor: Erlotinib [39]. This ability of miR-7 replacement to complement current treatment could increase response rates and efficacy and prolong survival. The effect of miRNA replacement to improve response rates has been shown using miR-34a in lung cancer, with increased survival times seen in mouse xenograft models of lung cancer treated with combined miRNA, siRNA and Cisplatin therapy [36]. As part of this study, we were unable to find any synergy action of combination Mitotane and miR-7 for cell proliferation in ACC cell lines (data not shown), but ongoing research in our lab suggest other miRNAs offer potential for synergistic treatment effects. It is with interest that in our study, that assessed single miRNA replacement, reduced tumor growth was achieved. This response may be further enhanced by using multiple miRNAs in a combinational approach [40]. Combination miRNA therapy has recently been assessed in a non-small lung cancer mouse model using two tumor suppressor miRNAs, resulting in an enhanced response compared to single miRNA replacement [41].

As this study was designed to investigate whether microRNA therapy has any utility for patients with metastatic ACC who have failed conventional treatment, miR-7 was delivered in a mouse xenograft model after the tumors were well formed. This design was used as given the experimental nature of microRNA therapy; any clinical trial will likely take place for patients with advanced disease. While our results showed xenograft stabilization or slowed xenograft growth, additional xenograft models using survival as an end-point or in an adjuvant model such as delivery of miRNAs at the time of tumor cells injection, would be of great interest to assess miRNA therapy as a novel treatment option in future studies.

In conclusion, miR-7 regulates ACC by targeting multiple cell pathways and miR-7 replacement therapy is effective in reducing ACC growth. The targeted systemic delivery approach of miR-7 replacement could be a useful treatment modality for multiple miR-7 deficient cancers. This study provides opportunities for future research for the role of miR-7 in complementing current therapeutics, combination miRNA therapy and further study of the possible role of miR-7 regulation of *CDK1* in cancer. As ACC has limited treatment options and a poor prognosis, ACC would make a meaningful model for clinical trials of miRNA replacement.

## MATERIALS AND METHODS

### Clinical samples

Ethics approval was obtained from the Northern Sydney Area Health Service Human Research Ethics Committee and informed consent was obtained from all patients whose samples were used in this study. ACC and NAC tissue samples were obtained during surgery, snap frozen in liquid nitrogen and stored at −80°C in the Neuroendocrine Tumor Bank of the Kolling Institute of Medical Research. The diagnosis of all samples was confirmed by centralized pathological review by an experienced endocrine pathologist.

### Cell culture and transfections

The human ACC cell line NCI-H295R (H295R) and SW13 were purchased from the American Type Culture Collection (ATCC, VA, USA). H295R cells (ATCC CRL-2128) were cultured in DMEM/F12 (Life Technologies, CA, USA) supplemented with 5% fetal bovine serum (FBS) and 1% ITS+ Premix supplement (BD Biosciences, MA, USA). SW-13 cells (ATCC CCL-105) were cultured in Leibovitz's medium (Life Technologies) supplemented with 10% FBS. Cells were cultured at 37°C in a humidified atmosphere under 5% CO_2_. The cell lines were negative in periodic monitoring for mycoplasma and independently genotyped to rule out cross-contamination by Cell Bank Australia (Westmead, Australia). Cells were transfected with a final concentration of 40 nM of synthetic miRNA mimics (*mir*Vana miRNA mimics, Cat No. 4464066 Life Technologies) corresponding to hsa-miR-7–5P (Product ID: MC10047) or a negative control miRNA (miR-NC: Product ID: AM171100) using Lipofectamine RNAiMax (Life Technologies) according to the manufacturer's protocol. For transfections, cells were plated in six-well plates and transfected when they were 50–70% confluent and three days later a second transfection was performed. Cells were collected for down-stream analysis three days after the second transfection.

### Cell proliferation and cell cycle analysis

Cell proliferation was performed using CellTiter 96 Aqueous One Solution Cell Proliferation Assay according to the manufacturer's instruction (MTS Assay, Promega, WI, USA). For this assay, 5,000 cells were cultured per well in a 96-well plate. Transfection of microRNA mimics were performed at the same time (measured as day 0) and proliferation data, using absorbance was measured on day 1 to day 5 following transfection. Absorbance at 490 nm was measured using a 96-well plate reader (Sunrise microplate reader, Tecan, Switzerland). For cell cycle analysis, 2.5 × 10^5^ H295R or SW-13 cells were cultured in a 6-well plate. Cells were collected, washed with Phosphate Buffered Saline (PBS) and stained with Propidium Iodide (PI, Sigma Aldrich) at a final concentration of 17.4 μg/ml. Cells were analysed using fluorescence-activated cell sorting (FACS) analysis (FACS Calibre, BD Biosciences) and flow cytometry histograms were modelled using Modfit LT software (Verity Software House, ME, USA).

### RNA extraction and RT-qPCR

Total RNA was extracted from frozen tumor samples and ACC cells using the miRNeasy Mini Kit (Qiagen, Hilden, Germany). RNA concentration and quality was assessed using a NanoDrop ND 1000 Spectrophotometer (ThermoFisher Scientific, MA, USA) and an Agilent 2100 Bioanalyzer (Agilent Technologies, CO, USA). The expression levels of individual miRNAs were measured with quantitative reverse transcription-polymerase chain reaction (RT-qPCR) using Taqman miRNA assays (Life Technologies) according to the manufacturer's instructions. Briefly, 10 ng of total RNA was first reverse transcribed to complementary DNA (cDNA) using TaqMan miRNA primers and the PCR products were then amplified from cDNA and quantified with the ABI 7900HT Real-time PCR System (Applied Biosystems) under standard cycling conditions. Relative expression (RQ) was obtained using the ΔΔC_t_ method and the differences between groups were assessed using DataAssist Version 3.01 (Applied Biosystems). RNU48 was used as a reference gene for human and xenograft samples and mouse U6 snRNA for mouse organs. Samples across all PCR plates were calibrated against commercially available human adrenal cortex total RNA (Clontech, CA, USA). For the measuring of mRNA expression levels, 1 μg of total RNA was reversed transcribed using the high capacity RNA-to-cDNA reverse transcription kit (Life Technologies) and the PCR was amplified using standard TaqMan gene expression assays (Life Technologies). *GAPDH* was used as a reference gene for human and xenograft samples and mouse *B2m* for mouse organs.

### Protein extraction and immunoblotting

Cells were lysed using Radio-Immunoprecipitation Assay (RIPA) Buffer and protein concentration was quantified using Pierce BCA Protein Assay Kit (Pierce Biotechnology, IL, USA). 30 μg of protein lysate was denatured at 70°C for 10 min before electrophoresis on precast 4–12% bis-Tris gels (Life Technologies). Separated proteins were transferred to Immobilin P membranes (Merck Millipore, MA, USA). The membranes were blocked in Tris-buffered saline with 0.1% Tween-20 (TBST) containing 5% BSA and probed with the antibody of interest. The Western Bright Quantum detection kit (Advansta, CA, USA) was used to visualize the detected proteins by a LAS4000 digital imaging system (Fujifilm, Tokyo, Japan). Protein loading was normalized to GAPDH and expression quantified using MultiGauge software (V 3.0, Fujifilm) and mean expression was calculated from three experiments.

### Luciferase reporter assays

Human genomic DNA was used to amplify the 3′ UTR of miRNA target genes by PCR and the amplified PCR fragment was cloned into the pMIR-REPORT Luciferase Vector (Part Number AM5795, Life Technologies) between *SpecI* and *SacI* restriction sites. For the EGFR-3′ UTR reporter, primers 5′-GACTACTAGTCTTCAATGGGCTCT TC CAACAAGG-3′ and 5′-GACTGAGCTCGGTCCAAA TGCTGATGAATCC-3′ were used to amplify a fragment of 532 bp containing two predicted miR-7 seed binding sequences. For the RAF1–3′ UTR reporter, primers 5′-GACTACTAGTGAAGTAAGGTAGCAGGCAGTCC-3′ and 5′-GACTGAGCTCTGAGGGACCATCAGATAAC TG-3′ were used to amplify a fragment of 555 bp also containing two seed binding miR-7 target sequences. ACC cells were co-transfected with the pMIR Luciferase Reporter Vector plus *Renilla* Luciferase Control Vector (Promega) along with the miRNA using Lipofectamine 2000 (Life Technologies) and the luciferase activity was quantified using the Dual-Luciferase Reporter Assay System (Promega) using a luminometer (Veritas Microplate Luminometer, Turner Biosystems, CA, USA). Relative luciferase activity was quantified by calculating the firefly to *Renilla* luciferase signal ratio.

### EDV^TM^ nanocell preparation

miRNAs were packaged into the EDVs for systemic delivery using a method of diffusion with overnight incubation previously reported for siRNA loading [42]. Following miRNA loading, EDVs were incubated with 5 μg of a bispecific monoclonal antibody (BsAb) against human EGFR for an hour at 24°C as previously reported [42]. The ensuing product was named ^EGFR^ EDV^TM^_miRNA_ (e.g. ^EGFR^ EDV^TM^_miR-7_).

### Mouse xenografts

Protocols for xenograft experiments in female athymic (*nu/nu*) mice (4–6 weeks old) were approved by the EnGeneIC Animal Ethics Committee. H295R cells (1 × 10^7^ cells in 100 μl serum free DMEM/F12K medium) with 100 μl BD Matrigel basement membrane matrix-growth factor reduced, phenol red free (BD Biosciences), which contains less than 0.5 ng/ml of epidermal growth factor, were inoculated subcutaneously into the left flank of each nude mouse. Patient-derived xenografts were established with inoculation of the same number of primary ACC cells isolated from ACC surgical tumor samples using the same protocol.

Mice were randomized to six mice per group to receive a control: scrambled miRNA sequences (^EGFR^EDV^TM^_miR-NC_) or treatment: miR-7 (^EGFR^EDV^TM^_miR-7_) when tumors reached ~100 mm^3^. Mice were treated four times per week by tail vein injection. Experiments ended when a significant difference between the treatment and control groups was detected according to the ethics protocol. Initial sample size was estimated by assuming a difference of 30% in tumor size between the control and treatment groups, with statistical significance of less than 0.05, a minimum of six animals were needed for greater than 90% power.

### Histopathology & immunohistochemistry

Following euthanasia, the tumors and organs were excised and flash frozen in liquid nitrogen and stored at −80°C. Histopathology on formalin fixed samples following H&E staining and immunohistochemistry was performed by a pathologist blinded to the treatment group. For EGFR staining, immunohistochemistry was scored semi-quantitatively from 0 (negative), to 1 + (focally or weakly positive) to 2 + (moderate staining) to 3 + (diffuse strong staining).

### Statistics

Statistics were calculated using Prism Software Version 6.0 (GraphPad, CA, USA) using Student's *t*-test for parametric data and Mann-Whitney test for nonparametric data. Differences in gene expression were assessed by *t*-test using DataAssist Version 3.01 (Applied Biosystems). Statistical significance was set as *P* ≤ 0.05.

## SUPPLEMENTARY FIGURE AND TABLE


