# Supplementary material for: microRNA-7 as a tumor suppressor and novel therapeutic for adrenocortical carcinoma
Source: Oncotarget. 2015 Oct 1;6(34):36675–88. doi: 10.18632/oncotarget.5383 (PMC4742203; doi:10.18632/oncotarget.5383)
Supplement: Supplementary file 1 [file oncotarget-06-36675-s001.pdf]

SUPPLEMENTARY FIGURE AND TABLE

RAF1:

```
      3'    uguuguuuuagugaUCAGAAGGu     5' hsa-miR-7-5p
                          ||||||||
  637:5'    cccucucagggagcAGUCUUCCa     3' RAF1 3'UTR

      3'    uguUGUUUUAGUGAUCAGAAGGu     5' hsa-miR-7-5p
               :| :|||  :| |||||||
  661:5'    cauGCUGAAU--UUUGUCUUCCa     3' RAF1 3'UTR
```

EGFR:

```
      3'    ugUUGU-UUUAGUGAUCAGAAGGu    5' hsa-miR-7-5p
              |:|| ||: ||| ||||||||
  441:5'    ggAGCACAAGCCACAAGUCUUCCa    3' EGFR 3'UTR

      3'    ugUUGUUUUAGUGAUCAGAAGGu     5' hsa-miR-7-5p
            :|| :| |:::| |||||||
  725:5'    uaGACUGACUUGUUUGUCUUCCa     3' EGFR 3'UTR
```

Schematic of two putative miR-7 seed binding sites in the *RAF1* and *EGFR* 3 UTR, respectively.

Supplementary Figure S1: Putative miR-7 seed binding sites in *RAF1* and *EGFR* 3′UTR.

Supplementary Table S1: Selected predicted targets for experimental validation

Target	Pathway
MTOR	*MTOR signalling*
EIF4E	*MTOR signalling*
PIK3CD	*MTOR signalling*
RPS6KB1	*MTOR signalling*
RAF1	*MAPK signalling*
IGF1-R	*Akt signalling*
EGFR	*MAPK signalling*
IRS2	*Insulin signalling*
RB1	*Cell Cycle signalling*
SP1	*TGF-Beta signalling*
